# The CSP (Cardiogenic Shock Prognosis) Score: A Tool for Risk Stratification of Cardiogenic Shock

**DOI:** 10.3389/fcvm.2022.842056

**Published:** 2022-03-07

**Authors:** Yu-Tzu Tien, Wen-Jone Chen, Chien-Hua Huang, Chen-Hsu Wang, Wei-Ting Chen, Chi-Sheng Hung, Jr-Jiun Lin, Ching-Chang Huang, Wei-Tien Chang, Min-Shan Tsai

**Affiliations:** ^1^Department of Emergency Medicine, National Taiwan University Medical College and Hospital, Taipei City, Taiwan; ^2^Department of Internal Medicine, National Taiwan University Hospital, Taipei City, Taiwan; ^3^Medical Intensive Care Unit, Cathay General Hospital, Taipei City, Taiwan

**Keywords:** cardiogenic shock, hospital mortality, nomogram, risk factors, prognosis, cardiogenic shock prognosis score

## Abstract

**Background:**

Cardiogenic shock (CS) is a critical condition and the leading cause of mortality after acute myocardial infarction (AMI). Scores that predict mortality have been established, but a patient's clinical course is often nonlinear. Thus, factors present during acute care management may be explored. This study intended to develop a risk-predictive model for patients with CS.

**Methods:**

In this observational study, adult patients who received inotropic support at the Emergency Room (ER) from January 2017 to August 2020 and were admitted to the cardiac care unit (CCU) with a diagnosis of CS were enrolled in this study. Patients with out-of-hospital cardiac arrest, inotropic support for bradycardia, and survival <24 h after ER arrival were excluded. A total of 311 patients were enrolled and categorized into derivation (*n* = 243) and validation (*n* = 68) cohorts.

**Results:**

A history of coronary artery disease, multiple inotrope use, ejection fraction <40%, lower hemoglobin concentration, longer cardiopulmonary resuscitation duration, albumin infusion, and renal replacement therapy were identified as independent prognostic factors for in-hospital mortality. The cardiogenic shock prognosis (CSP) score was established as a nomogram and three risk groups were identified: low-risk (score 115, 0% of mortality), medium-risk (score 116–209, 8.75% of mortality), and high-risk (score 210, 66.67% of mortality). The area-under-the-curve (AUC) of the CSP score was 0.941, and the discrimination value in the validation cohort was consistent (AUC = 0.813).

**Conclusions:**

The CSP score represents a risk-predictive model for in-hospital mortality in patients with CS in acute care settings. Patients identified as the high-risk category may have a poor prognosis.

## Introduction

Cardiogenic shock (CS) is the most severe form of acute heart failure and as a state of ineffective cardiac output, it results in clinical and biochemical manifestations of inadequate tissue perfusion (1). CS complicates up to 10% of cases of acute myocardial infarction (AMI) and is a leading cause of mortality after AMI (2). Despite advances in treatment options, CS mortality remains high at ~35–50% and is a challenging condition to manage in acute care settings (1, 2).

Several risk scores that help predict short-term mortality have been established (3–5). The SHOCK score and Intraaortic Balloon Pump in Cardiogenic Shock II trial (IABP-SHOCK II) trial were developed based on patients with MI and shock (3, 4). The CardShock study enrolled patients with all etiologies of CS, but more than half were acute CS (ACS) cases (5). The epidemiology of shock has evolved in recent years with AMI-related CS (AMICS) accounting for less than one-third of all CS cases, hence the role of hemodynamic stabilization using pharmacologic and nonpharmacologic therapies has been inconsistent (6, 7). All these risk scores revealed modest prognostic accuracy, with an internal validation area under the curve (AUC) of.74, 0.79, and.71, respectively (3–5).

The risk factors included in these models were mostly from the medical history and biochemistry results at admission; (3–5) however, CS is a critical condition that is ever-evolving from pre-shock to refractory shock states. Thus factors during a patient's acute care management may also affect the prognosis (2, 8). Besides, optimal management of CS requires timely interventions to prevent multiorgan system dysfunction (6). Early classification of CS may be needed to stratify illness severity to provide appropriate treatments and improve outcomes. Therefore, this study aimed to develop a risk-predictive model of in-hospital mortality for CS patients from varied etiologies, based on their medical history, examination results, and interventions during the early period of acute care to aid physicians in risk stratification and prognostication.

## Methods

### Study Design

This retrospective study was conducted in a tertiary hospital with 110,000 annual emergency room (ER) visits. The Institutional Review Board of National Taiwan University Hospital (NTUH) approved this study (202001104RINC).

### Study Population

**Figure 1:**
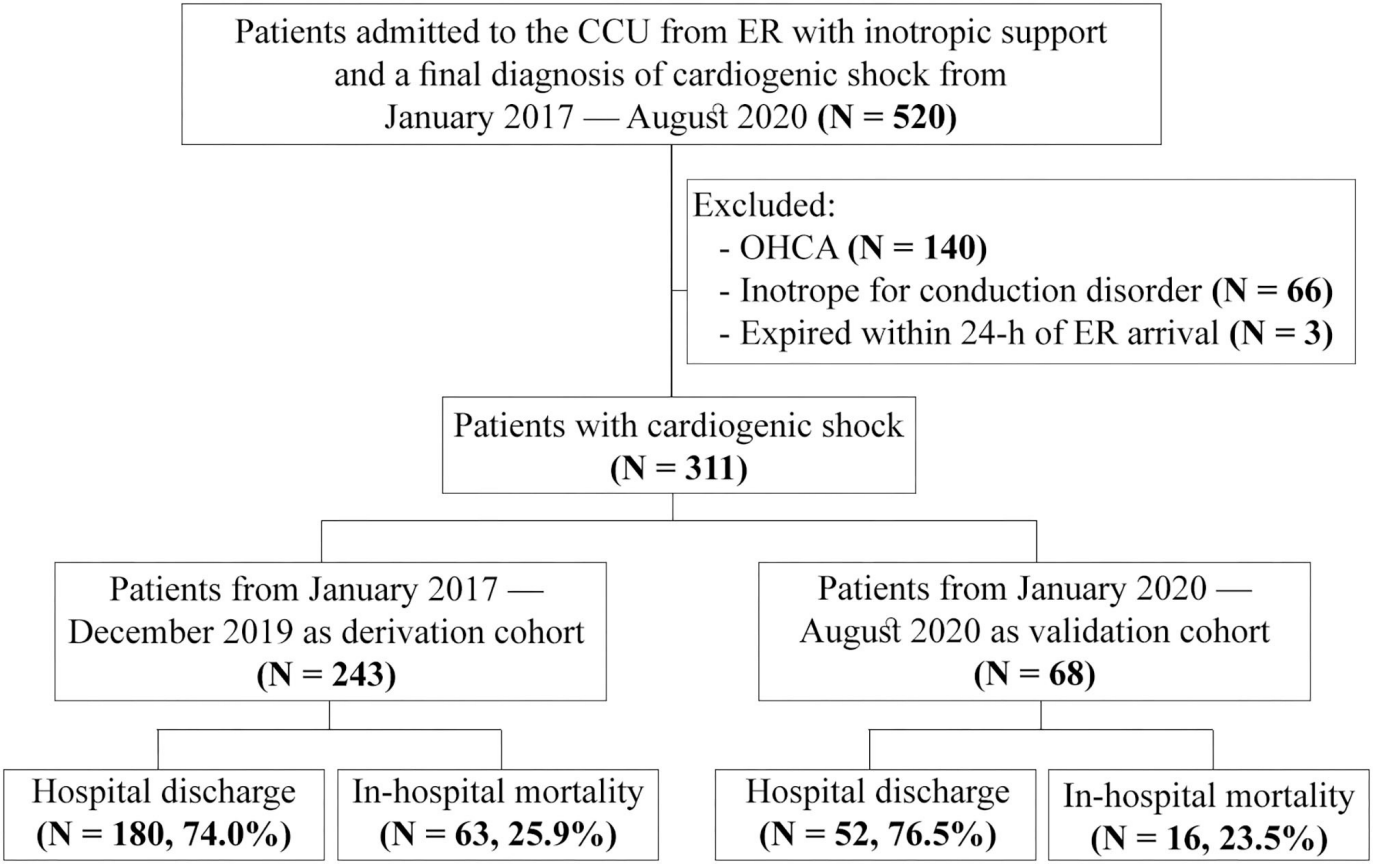
Flowchart of patient enrollment. CCU, cardiac care unit; ER, emergency room; OHCA, out-of-hospital cardiac arrest.

This study enrolled 520 non-traumatic adult patients (≥20 years old) who received inotropic support at the ER and subsequent admission to the cardiac care unit (CCU) with a diagnosis code of cardiogenic shock from January 2017 to August 2020. A total of 140 patients who had an out-of-hospital cardiac arrest (OHCA), 66 who received inotropic support for bradycardia and conduction system disorders, and 3 with survival <24 h after ER arrival were excluded. Finally, 311 patients were included in this study and categorized into the derivation cohort (*n* = 243, January 2017 – December 2019) and the validation cohort (*n* = 68, January 2020–August 2020) based on the timing of ER visits to develop the predictive model (Figure 1).

### Measurements

The following information was collected from the individual medical records: age, sex, preexisting comorbidities, clinical findings, laboratory and imaging exams nearest to the shock time at the ER, medications administered and clinical management at the acute care settings, discharge diagnosis, and length of hospital stay.

Vital signs were taken at the triage. Unconscious patients were defined if there was acute consciousness change on admission as documented on the medical record by the physician or the motor component of the Glasgow Coma Scale (GCS) has a score <6. Multiple inotrope use was the need for two or more of dopamine, norepinephrine, epinephrine, vasopressin, or dobutamine simultaneously to achieve hemodynamic stability. Patients who only required room air, nasal cannula, oxygen masks, or nonrebreathing masks were classified as having low respiratory support; whereas patients who required bilevel positive airway pressure (BiPAP), high flow oxygen therapy, or endotracheal intubation were classified as having high respiratory support.

The fluid challenge was considered when there was an infusion of more than 250 mL of a crystalloid before other interventions during shock. Coronary angiography (CAG) performed within 24 h of shock was considered emergent. Acute management for cardiogenic shock, when performed within 72 h of ER arrival, are as follows: cardiopulmonary resuscitation (CPR) attempt where CPR was performed at the ER or in the CCU; echocardiogram; renal replacement therapy (RRT) which encompassed patients who received dialysis, sustained low-efficiency dialysis (SLED), or continuous veno-venous hemofiltration (CVVH), after manifesting symptoms of fluid overload, respiratory distress, or severe electrolyte imbalance; transcutaneous pacing (TCP), referred as the noninvasive mode of temporary pacing by applying pads to the chest; pacemaker implantation, referred as the invasive method of inserting either temporary or permanent pacemakers (PPM); and the use of mechanical circulatory support (MCS) devices which included extracorporeal membrane oxygenation (ECMO) or intra-aortic balloon pump (IABP) insertion.

Discharge diagnoses were recorded based on the judgment of attending physicians, as documented in the medical discharge summary. Cases of STEMI, NSTEMI, and post-MI complications were classified as ACS; heart failure was classified as acute decompensated heart failure (ADHF); different types of cardiomyopathy (stress, restrictive, hypertrophic, dilated, ischemic) were classified as cardiomyopathy; tachyarrhythmia and bradyarrhythmia were classified as arrhythmia; CS with sepsis or pneumonia were classified as CS with septic complications; and valvular heart conditions, myocarditis, cardiac tamponade, aortic dissection, and pulmonary hypertension were considered as other causes.

### Outcome Measures

The primary outcome of this study was to identify predictors of in-hospital mortality of patients with CS. The secondary outcome was a risk-stratified predictive model and the validation of its performance with existing scores. The SHOCK score, IABP-SHOCK II risk score, and CardShock risk score were validated using the closest equivalent variable available from this study's dataset (Supplementary Table 1). Under the SHOCK score, unconscious patients were substituted for patients with anoxic brain damage, shock on admission was documented if the patient's systolic blood pressure (SBP) was <90 mmHg upon ER arrival, non-inferior MI was not included due to the difference in the study population, and end-organ hypoperfusion was not included due to limited data from medical records. Besides, non-inferior MI would garner no point if left ventricular ejection fraction (LVEF) was added to the scoring system, which was the case when obtaining the SHOCK score for validation. Data on glucose level and TIMI (Thrombolysis in Myocardial Infarction) flow grade after percutaneous coronary intervention (PCI) were not available for computing the IABP-SHOCK II trial score. For the CardShock risk score, unconscious patients were substituted for confusion at presentation.

### Statistical Data Analyses

Results are presented using frequencies for categorical variables and medians with quartiles for continuous variables. Fisher's exact or Pearson's chi-squared test for categorical variables and the Mann-Whitney U test for continuous variables were used for group comparisons. From the derivation cohort, 32 independent variables with significant associations (*p* < 0.05) and clinical importance in the univariate analysis were entered into the forward multiple logistic regression analysis (>0.1 for elimination) to identify the predictors of in-hospital mortality. The resulting variables from the multivariate analysis were then used to develop a risk-prediction nomogram. Discrimination was assessed with the AUC while calibration was evaluated with Hosmer-Lemeshow (HL) goodness-of-fit *x*^2^ estimates. Three risk groups for in-hospital mortality (low-, medium-, and high-risk) were defined by splitting the scoring system into tertiles of patients, patterned after Maupain et al. (9).

All statistical analyses were performed using SPSS Statistics for Windows, version 16.0 (SPSS Inc., Chicago, IL, USA), and R statistical software version 4.0.2 was used to construct the nomogram.

## Results

### Characteristics of Study Subjects

The baseline characteristics, laboratory, and imaging examinations of patients in the derivation cohort are presented in Table 1. The median age is 70 years and the majority of patients are men (60.16%). The incidence of coronary artery disease (CAD), heart failure, cardiomyopathy, and renal disease was determined to be higher in the patients who did not survive to discharge, whereas dyslipidemia was more frequent in the patients who survived. Echocardiograms with LVEF lower than 40% and valvular lesions were also observed more frequently in patients who failed to survive.

**Table 1:** Baseline characteristics and examination results between groups in the derivation cohort.

**Variables**	**Survival to discharge**			
	**Total patients**	**Survived**	**Non-survived**	***P-value***
	***N* = 243**	***N* = 180 (74.07%)**	***N* = 63 (25.92%)**	
Age (years)	70 (59–80)	71 (59–80.5)	70 (59–78)	0.566
Sex (male)	147 (60.5)	104 (57.8)	43 (68.3)	0.178
Clinical findings at triage				
SBP (mmHg)	100 (83–126)	102 (85–128)	94 (79–113)	0.021
Unconscious	39 (16.0)	23 (12.8)	16 (25.4)	0.027
Comorbidities				
Smoking	51 (21.0)	39 (21.7)	12 (19.0)	0.722
Alcoholism	9 (3.7)	5 (2.8)	4 (6.3)	0.243
Hypertension	126 (51.9)	96 (53.3)	30 (47.6)	0.466
Diabetes mellitus	88 (36.2)	62 (34.4)	26 (41.3)	0.362
Dyslipidemia	68 (28.0)	56 (31.1)	12 (19.0)	0.074
Old MI	10 (4.1)	8 (4.4)	2 (3.2)	0.739
CAD	77 (31.7)	49 (27.2)	28 (44.4)	0.013
Post CABG	12 (4.9)	10 (5.6)	2 (3.2)	0.529
Heart failure	65 (26.7)	38 (21.1)	27 (42.9)	0.001
Arrhythmia	67 (27.6)	47 (26.1)	20 (31.7)	0.415
Cardiomyopathy	25 (10.3)	14 (7.8)	11 (17.5)	0.034
Renal disease	37 (15.2)	22 (12.2)	15 (23.8)	0.040
ESRD	24 (9.9)	17 (9.4)	7 (11.1)	0.806
CVA	29 (11.9)	25 (13.9)	4 (6.3)	0.121
Malignancy	31 (12.8)	24 (13.3)	7 (11.1)	0.673
Laboratory exams				
pH value	7.35 (7.26–7.40)	7.35 (7.27–7.41)	7.31 (7.23–7.37)	0.062
Lactic acid (mmol/L)	3.38 (2.13–5.77)	3.05 (2.10–5.6)	5.26 (2.08–8.19)	0.000
Hemoglobin (g/dL)	12.70 (10.58–14.30)	13.10 (10.50–13.75)	11.80 (9.85–13.75)	0.021
Platelet (K/uL)	203 (156.50–266)	210 (153–253)	183 (140–266)	0.040
INR	1.08 (1.00–1.30)	1.06 (1.04–1.28)	1.27 (1.08–1.71)	0.000
Total bilirubin (mg/dL)	0.88 (0.65–1.99)	0.81 (0.62–1.54)	1.18 (0.69–3.87)	0.064
Creatinine (mg/dL)	1.65 (1.10–3.08)	1.50 (1.14–3.55)	2.20 (1.40–3.30)	0.001
eGFR	36 (18–59)	40 (21–65)	30 (14–43)	0.002
Sodium (mmol/L)	134 (130–137)	134.5 (130–137)	132 (127–134)	0.015
Potassium (mmol/L)	4.4 (3.7–5.1)	4.3 (3.6–5.4)	4.5 (3.7–5.1)	0.552
Troponin T (ng/L)	85.93 (28.91–370.20)	54.09 (28.18–283.15)	162.1 (85.82–399.40)	0.000
NT-proBNP (pg/mL)	4,932 (1,125–17,233)	3,470 (1,316–19,433)	13,415 (4,244–26,213)	0.000
ECG characteristics				
HR (bpm)	83 (54–108)	78 (64–116)	94 (76–111)	0.003
QRS duration (ms)	102 (88–138)	100 (92–149)	116 (97–146)	0.013
Chest X-ray				
Cardiomegaly	158 (65.0)	117 (65.0)	41 (65.1)	1.000
Lung edema	44 (18.1)	39 (21.7)	5 (7.9)	0.021
Pleural effusion	67 (27.6)	44 (24.4)	23 (36.5)	0.073
Echocardiogram				
LVEF <40%	81 (36.8)	47 (28.8)	34 (59.6)	0.000
Valvular lesions	117 (48.1)	78 (43.3)	39 (61.9)	0.013

*Data presented as no (%) or as median (IQR)*.

*CABG, coronary artery bypass grafting; CAD, coronary artery disease; CVA, cerebrovascular accident; eGFR, estimated glomerular filtration rate; ER, emergency room; ESRD, end-stage renal disease; INR, international normalized ratio; LVEF, left ventricular ejection fraction; old MI, old myocardial infarction; NT-proBNP, N-terminal-pro B-type natriuretic peptide; SBP, systolic blood pressure*.

Clinical management of patients who did not survive had received albumin infusion, multiple inotrope use, and heparin more frequently, as seen in Table 2. These patients also more frequently required high respiratory support, MCS devices, CABG surgery, component transfusion, and RRT; whereas pacemaker implantation was seen more in patients who survived. Patients who did not develop cardiac arrest had a better prognosis and among the patients with cardiac arrest following CS, resuscitation efforts were longer for patients who did not survive than for those who did.

**Table 2:** Treatments and diagnosis classification between groups in the derivation cohort.

**Variables**	**Survival to discharge**			
	**Total patients**	**Survived**	**Non-survived**	***P-value***
	***N* = 243**	***N* = 180 (74.0%)**	***N* = 63 (25.9%)**	
Medications				
Bronchodilator	93 (38.3)	62 (34.4)	31 (49.2)	0.050
Albumin	116 (47.7)	64 (35.6)	52 (82.5)	0.000
Diuretic	154 (63.4)	112 (62.2)	42 (66.7)	0.548
Inotrope use				
Single inotrope	116 (47.7)	112 (62.2)	4 (6.3)	0.000
Multiple inotropes	127 (52.3)	68 (37.8)	59 (93.7)	—
NTG	70 (28.8)	55 (30.6)	15 (23.8)	0.337
Aspirin	124 (51.0)	97 (53.9)	27 (42.9)	0.145
P2Y12 inhibitors	117 (48.1)	90 (50.0)	27 (42.9)	0.380
Heparin	146 (60.1)	97 (53.9)	49 (77.8)	0.001
Clinical management				
Respiratory support				
Low	99 (40.7)	93 (51.7)	6 (9.5)	0.000
High	144 (59.3)	87 (48.3)	57 (90.5)	—
Fluid challenge	96 (39.5)	68 (37.8)	28 (44.4)	0.372
Emergent CAG	119 (49.0)	93 (51.7)	26 (41.3)	0.188
PCI	76 (63.9)	59 (63.4)	17 (65.4)	0.291
CPR attempt (min)				
None	203 (83.5)	162 (90.0)	41 (65.1)	0.000
<10	18 (7.4)	13 (7.2)	5 (7.9)	—
10-20	6 (2.5)	1 (0.6)	5 (7.9)	—
>20	16 (6.6)	4 (2.2)	12 (19.0)	—
RRT	71 (29.2)	29 (16.1)	42 (66.7)	0.000
TCP	42 (17.3)	35 (19.4)	7 (11.1)	0.175
Pacemaker implantation	72 (29.6)	62 (34.4)	10 (15.9)	0.006
MCS	58 (23.9)	26 (14.4)	32 (50.8)	0.000
Component transfusion	134 (55.1)	79 (43.9)	55 (87.3)	0.000
CABG	16 (6.6)	7 (3.9)	9 (14.3)	0.008
Discharge diagnosis classification				
ACS	79 (32.5)	61 (33.9)	18 (28.6)	0.532
ADHF	58 (23.9)	39 (21.7)	19 (30.2)	0.229
Arrhythmia	56 (23.0)	49 (27.2)	7 (11.1)	0.009
Cardiomyopathy	15 (6.2)	10 (5.6)	5 (7.9)	0.545
Septic complication	7 (2.9)	3 (1.7)	4 (6.3)	0.076
Others	25 (10.3)	15 (8.3)	10 (15.9)	0.097
CCU stay (days)	5 (3–13)	4 (3–9)	14 (5–33)	0.000
Hospital stay (days)	12 (6–27)	12 (6–24)	14 (6–33)	0.580

*Data presented as no (%) or as median (IQR)*.

*ACS, acute coronary syndrome; ADHF, acute decompensated heart failure; CABG, coronary artery bypass grafting; CAG, coronary angiography; CCU, cardiac care unit; CPR, cardiopulmonary resuscitation; MCS, mechanical circulatory support devices; NTG, nitroglycerin; PCI, percutaneous coronary intervention; RRT, renal replacement therapy; TCP, transcutaneous pacing*.

The discharge diagnosis classification indicated that ACS was perceived as the main etiology of CS, followed by ADHF and arrhythmia. Patients with CS complicated by arrhythmia and septic complications had higher rates of survival than those who did not. Patients who did not survive had significantly longer CCU stays (Table 2). In-hospital mortality for patients with CS within 72 h from ER arrival was at 1.6% and upon discharge was at 25.9% (Supplementary Table 2).

The baseline characteristics, laboratory and imaging examinations, clinical management, and diagnosis classification of the validation cohort are presented in Supplementary Tables 3, 4. The median age is 72 years and the majority of such patients are men (61.76%). The ratios of patients per characteristic in the validation cohort were comparable to those in the derivation cohort. ACS remained the most common etiology of CS.

### Model Development and Validation

**Figure 2:**
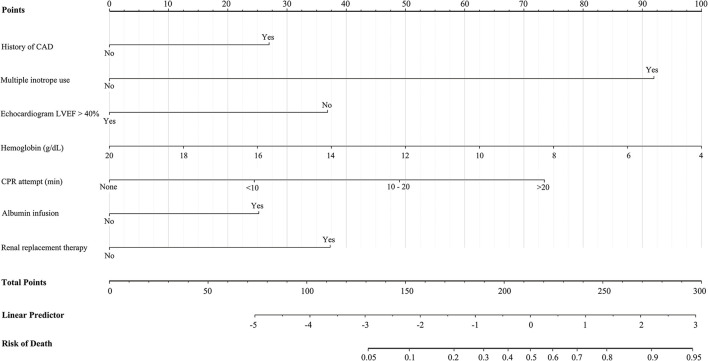
The Cardiogenic Shock Prognosis score, a predictive nomogram of in-hospital mortality for patients with cardiogenic shock. CAD, coronary artery disease; CPR, cardiopulmonary resuscitation; LVEF, left ventricular ejection fraction.

In the derivation cohort, 32 variables (SBP, unconscious, dyslipidemia, CAD, heart failure, cardiomyopathy, renal disease, lactic acid, hemoglobin, platelet, international normalized ratio (INR), creatinine, sodium, troponin, N-terminal-pro B-type natriuretic peptide (NT-proBNP), heart rate, QRS duration, lung edema, pleural effusion, LVEF, valvular lesions, bronchodilator use, albumin infusion, inotrope use, heparin use, respiratory support, CPR attempt, RRT, pacemaker implantation, MCS, component transfusion, and CABG) were identified from the univariate analysis and entered into the stepwise multiple logistic regression (Table 3). A history of CAD (OR 3.68, 95% CI 1.30–10.45, *p* =0.014), multiple inotrope use (OR 24.99, 95% CI 5.34–116.81, *p* < 0.001), LVEF <40% (OR 0.20, 95% CI 0.07–0.55, *p* = 0.002), low hemoglobin (OR 0.83, 95% CI 0.69–1.00, *p* = 0.053), albumin infusion (OR 4.74, 95% CI 1.49–15.14, *p* =0.009), CPR attempt (OR 2.21, 95% CI 1.23–3.97, *p* = 0.008), and RRT (OR 3.00, 95% CI 1.11–8.12, *p* = 0.031) remain associated with an increased risk for in-hospital mortality and a risk-prediction nomogram, the CSP (Cardiogenic Shock Prognosis) score, was developed accordingly (Figure 2). The CSP score allocated the individual prediction for having in-hospital mortality. For every patient, a virtual vertical line to the horizontal axis determined how many points should be attributed for each variable. Then, the total points provided a probability of in-hospital mortality.

**Table 3:** Adjusted and unadjusted odds ratios of predictive factors.

**Variables**	**Unadjusted odds ratio (95% CI)**	**Adjusted odds ratio (95% CI) Stepwise method**	***P*-value**
SBP (mmHg)	0.99 (0.98–1.00)	—	
Unconscious	2.32 (1.13–4.76)	—	
Dyslipidemia	0.52 (0.26–1.05)	—	
CAD	2.14 (1.18–3.88)	3.68 (1.30–10.45)	0.014
Heart failure	2.80 (1.52–5.18)	—	
Cardiomyopathy	2.51 (1.07–5.86)	—	
Renal disease	2.24 (1.08–4.66)	—	
Lactic acid (mmol/L)	1.20 (1.09–1.32)	—	
Hemoglobin (g/dL)	0.89 (0.80–0.98)	0.83 (0.69–1.00)	0.053
Platelet (K/uL)	1.00 (0.99–1.00)	—	
INR	2.36 (1.35–4.15)	—	
Creatinine (mg/dL)	1.11 (1.00–1.23)	—	
Sodium (mmol/L)	0.97 (0.92–1.01)	—	
Troponin T (ng/L)	1.00 (1.00–1.00)	—	
NT-proBNP (pg/mL)	1.00 (1.00–1.00)	—	
HR (bpm)	1.01 (1.00–1.01)	—	
QRS duration (ms)	1.01 (1.00–1.02)	—	
Lung edema	0.31 (0.12-0.83)	—	
Pleural effusion	1.78 (0.96–3.28)	—	
LVEF <40%	0.27 (0.146–0.51)	0.20 (0.07–0.55)	0.002
Valvular lesions	2.13 (1.18–3.83)	—	
Bronchodilator	1.84 (1.03–3.30)	—	
Albumin	8.57 (4.18–17.58)	4.74 (1.49–15.14)	0.009
Multiple inotrope use	24.29 (8.44–69.88)	24.99 (5.34–1,116.81)	0.000
Heparin	3.00 (1.54–5.81)	—	
Respiratory support	10.16 (4.17–24.74)	—	
CPR attempt	5.14 (2.56–10.35)	2.21 (1.23–3.97)	0.008
RRT	10.41 (5.40–20.10)	3.00 (1.11–8.12)	0.031
Pacemaker implantation	0.35 (0.17–0.75)	—	
MCS	6.11 (3.21–11.66)	—	
Component transfusion	8.79 (3.96-19.52)	—	
CABG	4.12 (1.47–11.58)	—	

*CABG, coronary artery bypass grafting; CAD, coronary artery disease; CI, confidence interval; CPR, cardiopulmonary resuscitation; INR, international normalized ratio; LVEF, left ventricular ejection fraction; MCS, mechanical circulatory support devices; NT-proBNP, N-terminal-pro B-type natriuretic peptide; RRT, renal replacement therapy; SBP, systolic blood pressure*.

**Figure 3:**
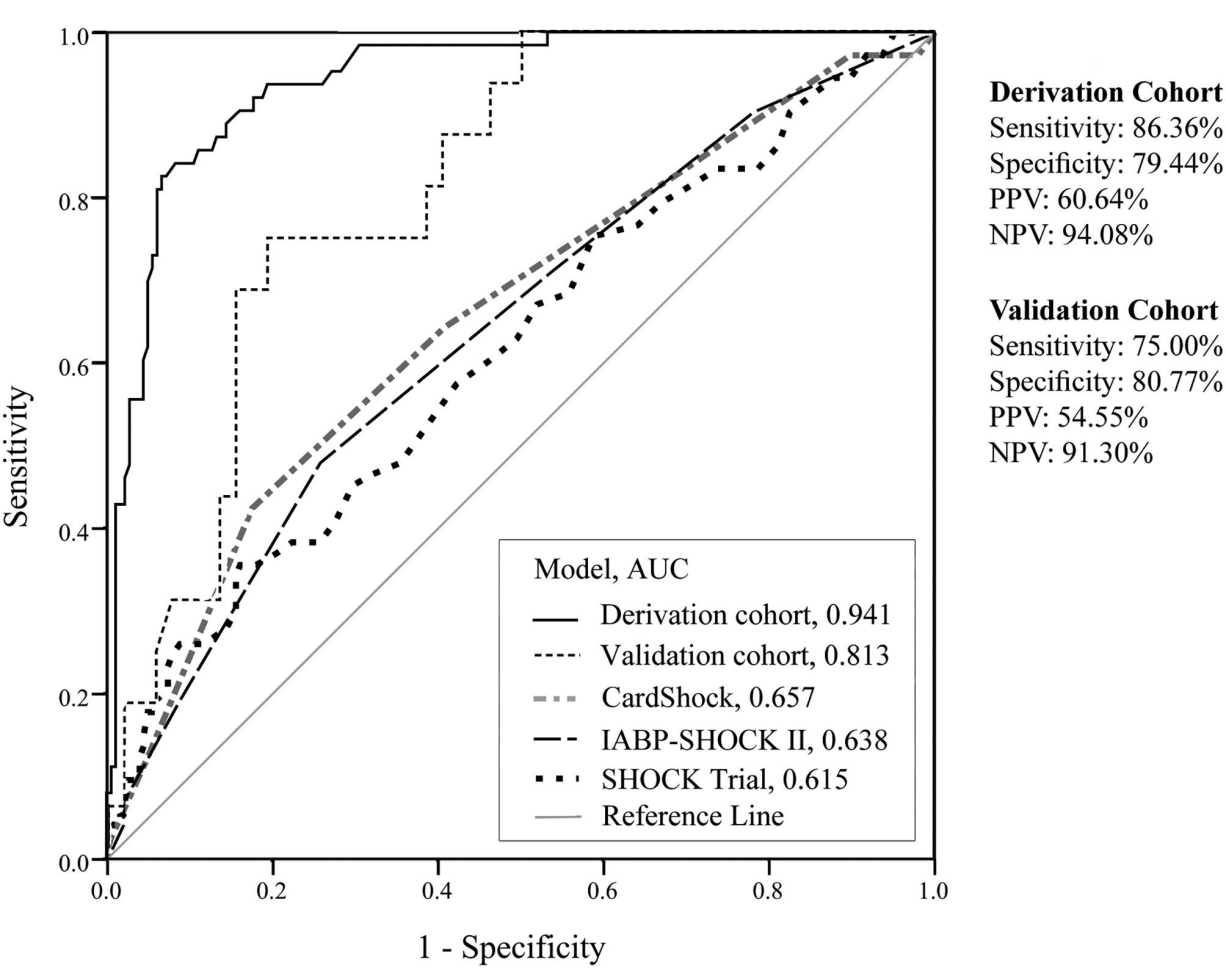
Discrimination performances of derivation cohort, validation cohort, and other scores. AUC, area under the curve; NPV, negative predictive value; PPV, positive predictive value.

The CSP score of the derivation cohort yielded an AUC of 0.941 (95% CI 0.91–0.97), indicating a good ability to discriminate the outcome of mortality. The model had an adequate goodness of fit (HL *x*^2^ = 4.786 with 8 df, *p* = 0.780). Internal validation resulted in a sensitivity of 75%, a specificity of 80.77%, and an AUC of 0.813 (95% CI 0.71–0.92). A comparison with other risk scores using the CS population (n=311) revealed the following AUCs: SHOCK score (0.615), IABP-SHOCK II trial score (0.638), and CardShock risk score (0.657). The results of the receiver operating characteristic (ROC) curves are illustrated in Figure 3.

### Model Risk Stratification

The CSP score was further stratified into three risk categories for in-hospital mortality. A CSP score of <115 was considered low-risk with a sensitivity of 0 and 6.25% in the development and validation cohorts, respectively. On the other hand, a score of more than 210 was considered high-risk and was associated with a sensitivity and specificity of 88.89, 84.44, 68.75, and 80.77% in the development and validation cohorts, respectively (Table 4). This threshold identified around two-thirds of patients with a high-risk score for an unfavorable outcome (Supplementary Table 5).

**Table 4:** Discrimination performance of Cardiogenic Shock Prognosis score with mortality.

**Study sample**	**CSP score**	
	**Low-risk**	**High-risk**
	**≤115**	**≥210**
Derivation cohort (*n* = 243)		
Sensitivity (95% CI)	0 (0–7.16)	88.89% (77.84–95.04)
Specificity (95% CI)	56.11% (48.53–63.43)	84.44% (78.13–89.25)
Positive predictive value (95% CI)	0 (0–5.78)	66.67% (55.44–76.35)
Negative predictive value (95% CI)	61.59% (53.65–68.97)	95.60% (90.79–98.06)
Accuracy (95% CI)	41.56% (38.52–45.43)	85.60% (78.07–91.10)
Validation cohort (*n* = 68)		
Sensitivity (95% CI)	6.25% (0.33–32.39)	68.75% (41.48–87.87)
Specificity (95% CI)	48.08% (34.22–62.22)	80.77% (67.03–89.92)
Positive predictive value (95% CI)	3.57% (0.19–20.24)	52.38% (30.34–73.61)
Negative predictive value (95% CI)	62.50% (45.81–76.83)	89.36% (76.11–96.02)
Accuracy (95% CI)	38.24% (29.33–51.55)	77.94% (63.33–89.19)

*CSP, Cardiogenic Shock Prognosis*.

## Discussion

In this retrospective observational study, factors including a history of CAD, multiple inotrope use, LVEF <40%, lower hemoglobin concentration, albumin infusion, longer CPR attempt, and RRT were identified to be associated with increased in-hospital mortality among patients with cardiogenic shock. The CSP score, a risk-predictive nomogram, was developed with an intended predictive utility within 72 h of acute care or immediately after admission, and stratified patients into three risk groups with good performance.

Previous scoring systems mostly focused on cardiogenic shock secondary to ACS (3–5) but in recent years, a significant proportion of cases are due to other etiologies (7). As compared with patients with ACS etiology, non-ACS patients had a more favorable course (5). This study's focus to include cases with more heterogeneous causes of CS may be applicable for use in various populations. The evolving epidemiology of CS cases may mean approaches in managing patients with AMICS may not be as effective in treating patients with other CS etiologies (7). Hence, an important guide in clinical decision-making could be to first stratify patients according to mortality risk and adapt intervention strategies accordingly. This study developed the CSP score which classified CS patients into three risk groups: scores of <115 were determined to be low-risk (0% mortality), scores of 116–209 as medium-risk (8.75% mortality), and more than 210 as high-risk (66.67% mortality). The condition of patients with CS lies on a continuum, progressing from pre-shock states to severe shock states at different rates and requiring simultaneous interventions to maintain hemodynamic stability (2, 6). This study takes this into consideration and identified predictive factors for mortality risk within 72 h of acute care management or immediately after admission for more accurate prognostication.

Several mortality predictors have been identified in previous scoring systems. Among them, previous MI or CABG and reduced LVEF were risk factors based on the SHOCK (3) and CardShock risk scores (5), consistent with this study's history of CAD and reduced cardiac function. High creatinine levels or low eGFR were present in all three scores whereas the need for RRT during acute care was determined to be a risk factor in this study. Other predictors identified in this study but not reported in other scores include multiple inotrope use, albumin infusion, lower hemoglobin levels, and longer CPR duration.

These factors altogether contribute to the illness severity of patients with CS. Vasopressors and inotropes are the cornerstones of CS management but mortality was significantly higher with escalating use, and adrenaline being the most evident (10, 11). Albumin infusion for hypoalbuminemia is correlated with higher illness severity and can act as a frailty biomarker among patients with heart failure or ACS (12, 13). Among patients with ACS, studies have shown that lower hemoglobin levels on admission are an independent predictor of increased risk for short-term mortality, more so if complicated with comorbidities of hypertension or chronic renal disease (14, 15). When complicated with CS, a higher hemoglobin concentration is a protective factor for the development of in-hospital cardiac arrest (16). Lastly, cardiac arrest patients with a prolonged CPR duration were observed to be associated with a poorer prognosis (17, 18). Early stratification of these patients may guide clinician decision-making.

### Limitations

Several limitations of this study should be considered. First, the retrospective nature of the study caused unavoidable selection bias. Unrecognized confounding factors may be present. Second, the small sample size from a single center may have resulted in nonsignificant differences between groups in some variables. Besides, patients resuscitated from OHCA and without the survival of more than 24 h were excluded from the current study, therefore, patients with the most severe CS may not be evaluated. Third, this study is largely based on an Asian demographic and thus may be more applicable for similar populations. Certain hospitalization procedures may vary per country such as the availability of IABP and ECMO support may differ from the Western practices. Another difference in procedure is the preference for albumin infusion as a volume expander in shock patients after fluid challenge in Taiwan, which is covered by the National Health Insurance. Furthermore, the limited availability of variable substitutes from this study's dataset for validation of the SHOCK trial score, IABP-SHOCK II trial score, and CardShock risk score may have contributed to a better predictive performance in the CSP score and a lower mortality rate in this study. Thus, a larger sample size and external validation for the model are necessary before extrapolating it to other populations. Finally, this study focused on developing a risk-stratification tool for in-hospital mortality, therefore recommending effective interventions depending on risk severity or predicting long-term prognosis after discharge will require future investigation through well-designed studies.

## Conclusions

The CSP score which included a history of CAD, multiple inotrope use, ejection fraction <40%, lower hemoglobin concentration, longer CPR attempt, albumin infusion, and RRT was generated with high performances in predicting in-hospital mortality among CS patients in the acute care setting. The high-risk group (CSP score ≥ 210) showed a high sensitivity for poor prognosis.

## Data Availability Statement

The de-identified datasets used and analyzed during the study will be shared upon reasonable request.

## Ethics Statement

The studies involving human participants were reviewed and approved by Institutional Review Board of National Taiwan University Hospital (202001104RINC). Written informed consent for participation was not required for this study in accordance with the national legislation and the institutional requirements.

## Author Contributions

MST, CHH, WJC, and CHW contributed to the study concept and design. YTT, MST, CCH, CHW, CSH, WTChe, WTCha, and JJL contributed to the acquisition of the data. YTT and MST analyzed and interpreted the data and drafted the manuscript. CHH and WJC provided critical revision of the manuscript for important intellectual content and supervised the study. All authors contributed to manuscript revision, read, and approved the submitted version.

## Funding

This study was supported by the National Taiwan University Hospital, Taipei City, Taiwan, project 110-CGN05.

## Conflict of Interest

The authors declare that the research was conducted in the absence of any commercial or financial relationships that could be construed as a potential conflict of interest.

## Publisher's Note

All claims expressed in this article are solely those of the authors and do not necessarily represent those of their affiliated organizations, or those of the publisher, the editors and the reviewers. Any product that may be evaluated in this article, or claim that may be made by its manufacturer, is not guaranteed or endorsed by the publisher.
