# Supplementary material for: The CSP (Cardiogenic Shock Prognosis) Score: A Tool for Risk Stratification of Cardiogenic Shock
Source: Front Cardiovasc Med. 2022 Mar 7;9:842056. doi: 10.3389/fcvm.2022.842056 (PMC8936130; doi:10.3389/fcvm.2022.842056)
Supplement: Supplementary file 1 [file Data_Sheet_1.docx]

Supplementary Material

# Supplementary Figures and Tables

**Supplementary Table 1. Variable substitutes for validation with other scores**

| Risk Score | Variable | Variable Substitute* |
| --- | --- | --- |
| SHOCK trial | Anoxic brain damage | Unconscious patient |
|  | Shock on admission | SBP <90 mmHg at ER admission |
|  | Non-inferior MI | Not included; no point if LVEF was added |
|  | LVEF | — |
|  | Age | — |
|  | End-organ hypoperfusion | Not included |
|  | Prior CABG | — |
|  | Creatinine | — |
|  | SBP | — |
| IABP-SHOCK II | Age | — |
|  | History of stroke | — |
|  | Glucose | Not included |
|  | Creatinine | — |
|  | Arterial lactate | — |
|  | TIMI flow grade after PCI | Not included |
| CardShock | Age | — |
|  | Confusion at presentation | Unconscious patient |
|  | Previous MI or CABG | — |
|  | ACS etiology | — |
|  | LVEF | — |
|  | Blood lactate | — |
|  | eGFR | — |

*Variable substitutes were selected from the closest equivalent variable available from this study’s dataset.

ACS, acute coronary syndrome; CABG, coronary artery bypass grafting; eGFR, estimated glomerular filtration rate; LVEF, left ventricular ejection fraction; MI, myocardial infarction; PCI, percutaneous coronary intervention; SBP, systolic blood pressure; TIMI, Thrombolysis in Myocardial Infarction.

**Supplementary Table 2. Comparison of survival time between groups**

| Time after ER arrival | Derivation cohort  N = 243 | | Validation cohort  N = 68 | |
| --- | --- | --- | --- | --- |
|  | Survived  n (%) | Non-survived  n (%) | Survived  n (%) | Non-survived  n (%) |
| 48 hours | 240 (98.8) | 3 (1.2) | 66 (97.1) | 2 (2.9) |
| 72 hours | 239 (98.4) | 4 (1.6) | 66 (97.1) | 2 (2.9) |
| 7 days | 226 (93.0) | 17 (7.0) | 65 (95.6) | 3 (4.4) |
| Upon discharge | 180 (74.1) | 63 (25.9) | 52 (76.5) | 16 (23.53) |

Data presented as no. (%)

ER, emergency room.

**Supplementary Table 3. Baseline characteristics and examination results between groups in the validation cohort**

|  |  | Survival to Discharge | | |
| --- | --- | --- | --- | --- |
| Variables | Total Patients | Survived | Non-survived | *p-value* |
|  | N = 68 | N = 52 (76.47) | N = 16 (23.53) |  |
| Age (years) | 72 (64-80) | 72 (64-78) | 75 (70-82) | 0.050 |
| Sex (male) | 42 (61.76) | 32 (61.54) | 10 (62.50) | 0.945 |
| Clinical findings at triage | | | | |
| SBP (mmHg) | 94 (79-113) | 93 (81-106) | 100 (76-124) | 0.626 |
| Unconscious | 11 (16.18) | 6 (11.54) | 5 (31.25) | 0.061 |
| Comorbidities | | | | |
| Smoking | 2 (2.94) | 2 (3.85) | 0 | 0.426 |
| Alcoholism | 1 (1.47) | 1 (1.92) | 0 | 0.576 |
| Hypertension | 44 (64.71) | 34 (65.38) | 10 (62.50) | 0.833 |
| Diabetes mellitus | 29 (42.65) | 22 (42.31) | 7 (43.75) | 0.919 |
| Dyslipidemia | 25 (36.76) | 20 (38.46) | 5 (31.25) | 0.601 |
| Old MI | 1 (1.47) | 0 | 1 (6.25) | 0.069 |
| CAD | 23 (33.82) | 16 (30.77) | 7 (43.75) | 0.337 |
| Heart failure | 17 (25.00) | 13 (25.00) | 4 (25.00) | 1.000 |
| Arrhythmia | 17 (25.00) | 12 (23.08) | 5 (32.25) | 0.509 |
| Cardiomyopathy | 8 (11.76) | 5 (9.62) | 3 (18.75) | 0.321 |
| Renal disease | 17 (25.00) | 11 (21.15) | 6 (37.50) | 0.187 |
| ESRD | 5 (7.35) | 4 (7.69) | 1 (6.25) | 0.847 |
| CVA | 14 (20.59) | 10 (19.23) | 4 (25.00) | 0.618 |
| Malignancy | 6 (8.82) | 5 (9.62) | 1 (6.25) | 0.678 |
| Laboratory exams | | | | |
| pH value | 7.32 (7.23-7.38) | 7.33 (7.24-7.33) | 7.21 (7.11-7.25) | 0.952 |
| Lactic acid (mmol/L) | 3.32 (2.37-5.56) | 3.21 (3.07-6.66) | 4.62 (3.52-10.75) | 0.073 |
| Hemoglobin (g/dL) | 12.25 (9.85-113.85) | 12.20 (10.00-14.10) | 12.65 (9.85-13.45) | 0.614 |
| Platelet (K/uL) | 197 (142-251) | 200.50 (123-168) | 183 (132-213) | 0.270 |
| INR | 1.04 (1.00-1.26) | 1.04 (0.96-1.20) | 1.05 (1.08-2.01) | 0.378 |
| Total bilirubin (mg/dL) | 0.75 (0.55-1.16) | 0.73 (0.70-1.31) | 1.09 (0.76-1.41) | 0.077 |
| Creatinine (mg/dL) | 1.6 (1.20-2.58) | 1.7 (1.10-1.50) | 1.6 (1.25-3.90) | 0.768 |
| eGFR | 38 (17-55) | 36 (18-55) | 42 (16-54) | 0.894 |
| Sodium (mmol/L) | 135 (130-137) | 135 (135-140) | 134 (129-135) | 0.660 |
| Potassium (mmol/L | 4.4 (3.9-5.0) | 4.35 (3.1-4.5) | 4.6 (4.7-5.9) | 0.735 |
| Troponin T (ng/L) | 84.51 (35.90-401.93) | 75.61 (26.70-59.22) | 140.50 (92.35-5348.90) | 0.470 |
| NT-proBNP (pg/mL) | 3158 (748-17384) | 2864 (50-2738) | 16790 (8679-35000) | 0.039 |
| ECG characteristics | | | | |
| HR (bpm) | 84 (43-102) | 61 (82-114) | 88 (82-105) | 0.016 |
| QRS duration (ms) | 105 (91-148) | 103 (102-158) | 131 (118-158) | 0.026 |
| Chest X-ray | | | | |
| Cardiomegaly | 47 (69.12) | 32 (61.54) | 15 (93.75) | 0.015 |
| Lung edema | 19 (27.94) | 15 (28.85) | 4 (25.00) | 0.764 |
| Pleural effusion | 17 (25.00) | 9 (17.31) | 8 (50.00) | 0.008 |
| Echocardiogram | | | | |
| LVEF < 40% | 20 (34.48) | 12 (27.27) | 8 (57.14) | 0.041 |
| Valvular lesions | 45 (66.18) | 32 (61.54) | 13 (81.25) | 0.145 |

Data presented as no. (%) or as median (IQR).

CABG, coronary artery bypass grafting; CAD, coronary artery disease; CVA, cerebrovascular accident; eGFR, estimated glomerular filtration rate; ER, emergency room; ESRD, end-stage renal disease; INR, international normalized ratio; LVEF, left ventricular ejection fraction; old MI, old myocardial infarction; NT-proBNP, N-terminal-pro B-type natriuretic peptide; SBP, systolic blood pressure.

**Supplementary Table 4. Treatments and diagnosis classification between groups in the validation cohort**

|  |  | | Survival to Discharge | | | | |  |
| --- | --- | --- | --- | --- | --- | --- | --- | --- |
| Variables | Total Patients | | Survived | | Non-survived | | *p-value* |  |
|  | N = 68 | | N = 52 (76.47) | | N = 16 (23.53) | |  |  |
| Medications |  | |  | |  | |  |  |
| Bronchodilator | 15 (22.06) | | 8 (15.38) | | 7 (43.75) | | 0.017 |  |
| Albumin | 27 (39.71) | | 14 (26.92) | | 13 (81.25) | | 0.000 |  |
| Diuretic | 42 (61.76) | | 31 (59.62) | | 11 (68.75) | | 0.511 |  |
| Inotrope use |  | |  | |  | |  |  |
| Single inotrope | 32 (47.06) | | 29 (55.77) | | 3 (18.75) | | 0.009 |  |
| Multiple inotropes | 36 (52.94) | | 23 (44.23) | | 13 (81.25) | | — |  |
| NTG | 14 (20.59) | | 11 (21.15) | | 3 (18.75) | | 0.835 |  |
| Aspirin | 32 (47.06) | | 24 (46.15) | | 8 (50.00) | | 0.788 |  |
| P2Y12 inhibitors | 33 (48.53) | | 26 (50.00) | | 7 (43.75) | | 0.662 |  |
| Heparin | 39 (57.35) | | 27 (51.92) | | 12 (75.00) | | 0.103 |  |
| Clinical management |  | |  | |  | |  |  |
| Respiratory support |  | |  | |  | |  |  |
| Low | 33 (48.53) | | 30 (57.69) | | 3 (18.75) | | 0.006 |  |
| High | 35 (51.47) | | 22 (42.31) | | 13 (81.25) | | — |  |
| TCP | 7 (10.29) | | 6 (11.54) | | 1 (6.25) | | 0.543 |  |
| Pacemaker implantation | 22 (32.35) | | 21 (40.38) | | 1 (6.25) | | 0.011 |  |
| Fluid challenge | 30 (44.12) | | 23 (44.23) | | 7 (43.75) | | 0.973 |  |
| Component transfusion | 35 (51.47) | | 20 (38.46) | | 15 (93.75) | | 0.000 |  |
| MCS | 18 (26.47) | | 10 (19.23) | | 8 (50.00) | | 0.015 |  |
| CPR attempt (min) |  | |  | |  | |  |  |
| None | 50 (73.53) | | 43 (82.69) | | 7 (43.75) | | 0.011 |  |
| <10 | 10 (14.71) | | 5 (9.62) | | 5 (31.25) | | — |  |
| 10-20 | 1 (1.47) | | 1 (1.92) | | 0 | | — |  |
| >20 | 7 (10.29) | | 3 (5.77) | | 4 (25.00) | | — |  |
| Emergent CAG | 35 (51.47) | | 28 (53.85) | | 7 (43.75) | | 0.480 |  |
| PCI | 17 (25.00) | | 12 (23.08) | | 5 (31.25) | | 0.509 |  |
| CABG | 7 (10.29) | | 4 (7.69) | | 3 (18.75) | | 0.203 |  |
| RRT | 23 (33.83) | | 13 (25.00) | | 10 (62.50) | | 0.006 |  |
| Discharge diagnosis classification | |  | |  | |  | | |
| ACS | 21 (30.88) | 15 (28.85) | | 6 (37.50) | | 0.512 | | |
| ADHF | 13 (19.12) | 8 (15.38) | | 5 (31.25) | | 0.158 | | |
| Arrhythmia | 18 (26.47) | 17 (32.69) | | 1 (6.25) | | 0.036 | | |
| Cardiomyopathy | 3 (4.41) | 2 (3.85) | | 1 (6.25) | | 0.682 | | |
| Septic complication | 0 | 0 | | 0 | | — | | |
| Others | 11 (16.18) | 8 (15.38) | | 3 (18.75) | | 0.749 | | |
| CCU stay (days) | 5 (3-12) | 4 (4-13) | | 15 (9-17) | | 0.003 | |  |
| Hospital stay (days) | 12 (7-24) | 12 (12-63) | | 17 (9-17) | | 0.433 | |  |

Data presented as no. (%) or as median (IQR).

ACS, acute coronary syndrome; ADHF, acute decompensated heart failure; CABG, coronary artery bypass grafting; CAG, coronary angiography; CCU, cardiac care unit; CPR, cardiopulmonary resuscitation; MCS, mechanical circulatory support devices; NTG, nitroglycerin; RRT, renal replacement therapy; TCP, transcutaneous pacing.

**Supplementary Table 5. Outcomes of groups stratified by CSP severity.**

| Study Sample | CSP Score | | | |
| --- | --- | --- | --- | --- |
|  | Total | Low-risk  ≦ 115 | Medium-risk  116 – 209 | High-risk  ≧ 210 |
| Derivation cohort | | | | |
| Patient number, n | 243 | 79 | 80 | 84 |
| In-hospital mortality, n (%) | 63 | 0 | 7 (8.75) | 56 (66.67) |
| Validation cohort | | | | |
| Patient number, n | 68 | 28 | 19 | 21 |
| In-hospital mortality, n (%) | 16 | 1 (3.57) | 4 (21.05) | 11 (52.38) |

CSP, Cardiogenic Shock Prognosis.
